# Ensuring that a school-based smoking cessation program for adolescents is successful: A realist evaluation of the TABADO program and the program theory

**DOI:** 10.1371/journal.pone.0283937

**Published:** 2023-04-06

**Authors:** Amandine Vallata, François Alla

**Affiliations:** 1 INSERM, BPH, U1219, I-prev/PHARES, Equipe Labellisée Ligue Contre le Cancer, CIC 1401, Univ. Bordeaux, Bordeaux, France; 2 Département d’Addictologie, Pôle Hospitalo-Universitaire de Psychiatrie d’Adultes et d’Addictologie du Grand Nancy, Centre Psychothérapique de Nancy, Laxou, France; 3 CHU de Bordeaux, Prevention Department, Bordeaux, France; University College London, UNITED KINGDOM

## Abstract

**Background:**

A smoking cessation program for adolescents, TABADO, demonstrated its effectiveness following a controlled trial conducted in 2007/2009. The program is now being scaled up nationally. In order to retain its efficacy across the diversity of contexts in the generalization process, we needed to assess the processes and mechanisms that inform its effects. Theory-driven evaluation is one approach used to address these issues. The aim of the present research is to develop the TABADO program theory. More specifically, we attempt to identify the factors and mechanisms that promote or hinder the enrollment and retention of student smokers in the program.

**Methods:**

We conducted a realist evaluation of the TABADO program through 1) a documentary analysis to construct the initial program theory, and 2) a multiple case study (n = 10) conducted in three regions in France to test and enrich the initial theory with contextual, organizational and mechanistic components. We used the Intervention-Context-Actors-Mechanisms-Outcomes configurations to guide our analysis and to present our results.

**Results:**

Our analysis highlighted 13 mechanisms that foster the enrollment and retention of student smokers in the TABADO program (e.g., being prepared to quit smoking, feeling encouraged in the attempt to quit smoking). To activate these mechanisms, the involvement of various actors is required (e.g., the school nurse, teachers), together with a combination of interventional and contextual factors (e.g., confidentiality, informal speaking time).

**Conclusions:**

These findings allowed us to transform the TABADO program into a new optimized strategy, TABADO2, which is theory-based. Our research helps to explain why adolescent smokers enroll and stay in a school-based smoking cessation program. TABADO2 needs to be considered in a more comprehensive way than the original research-based TABADO, and should be tailored to its implementation context.

## 1 Introduction

To address the major issue of adolescent smoking, a school-based cessation program was launched in France, the TABADO program [1]. It was evaluated in 2007/09 through a quasi-experimental controlled cluster study, which demonstrated its effectiveness. The adjusted one-year cessation rate was twice as high in schools benefiting from the TABADO program compared to the control schools (17% vs. 11.9%, adjusted OR = 2.1 [1.2 to 3.6], p = 0.008) [2]. The program, which is now referenced in the Santé publique France evidence-based intervention register [3], is being scaled-up nationally by the Institut national du cancer [4]. In 2020–2021, 215 institutions in 14 regions were expected to roll out the program to 77,000 young people [5].

The TABADO program is a complex intervention [6–8], because its outcome is the result of an interaction between interventional and contextual components. Its real-life implementation and generalization across a diversity of contexts to demonstrate its effectiveness in a specific experimental context is not self-evident in terms of applicability and transferability [9]. For such complex interventions, we need to go beyond the evaluation of effectiveness to assess the underlying processes and mechanisms [7, 10]. The identification of these elements allows us to make recommendations in terms of transferability and scaling up.

Theory-driven evaluation is an approach that can be used to address these issues. Its central concept is that of program theory [11]. Two main types of theory can be identified: those focused primarily on implementation, answering the question "how?", and those focused primarily on explaining causality, answering the question "why?" [12, 13]. In this study, we consider program theory as a combination of these two concepts. Realist evaluation offers one possible approach to identifying the theory of a complex intervention [7, 14].

The overall aim of this work is to develop the TABADO program theory. More specifically, we attempt to identify the factors and mechanisms that promote or hinder the enrollment and retention of student smokers in this program, which has already proved its efficacy.

From a pragmatic point of view, the answers to this scientific question also allow us to put forward recommendations to accompany the national scale-up process [3, 4].

## 2 Methods

### 2.1 Description of the TABADO program

TABADO was developed as a smoking cessation program for vocational schools, which was accessible, free of charge and completely confidential [1]. It is based on a combination of pharmaceutical treatments and psychological support and includes [2]:

- An information session on tobacco in whole classes for smokers and non-smokers. At the end of the session, students who smoke are given the chance to join the TABADO program to help them stop smoking.
- The volunteers then join a free-of-charge support program directly in the school. It includes an initial individual diagnostic consultation with a tobaccologist, then a monthly follow-up consultation for four months, accompanied by a small group session with the other volunteers enrolled in the program. Nicotine substitutes are given free of charge by the tobaccologist if they are deemed necessary.

The techniques used during the individual consultations and group sessions are inspired by cognitive-behavioral therapies, designed to reinforce the motivation to quit and to encourage peer support.

### 2.2 Study design

This study uses the RAMESES II standards as recommended for reporting realist evaluations (S1 Table) [15].

The method adopted is that of realist evaluation, from Pawson and Tiley [14, 16]. It is based on the relationship between three elements, called CMO configurations: contextual conditions (C), which will activate certain mechanisms (M), which in turn produce outcomes (O). In this work, we retained the following definition of mechanism: "A mechanism is hidden but real, is an element of reasoning and reactions of agents in regard to the resources available in a given context to bring about changes through the implementation of an intervention, and evolves within an open space-time and social system of relationships." [17]. To clarify the contextual conditions, some authors distinguish between elements relating to the Intervention (I), the Context (C) and the Actors (A), giving "ICAMO" configurations [18–21], which we have retained here. The set of ICAMOs is formed by identifying “regularities”, that is, repetitive factors among different situations, and constitutes the program theory.

In our realist approach, we followed the following three steps (S1 Fig) [16]: 1) identify the initial theory underlying the construction of the first TABADO, 2) collect data from various TABADO implementation contexts to test and enrich the initial hypotheses, and 3) in view of these new elements, develop ICAMO configurations and create an enriched theory.

### 2.3 Initial program theory

The initial theory underlying the TABADO program was not formalized by the intervention designers. A documentary analysis was conducted on the TABADO research protocol (internal unpublished document) and on published articles [1, 2, 22]. This analysis was supplemented by interviews with one of the TABADO designers. The aim was to identify the main components of the intervention together with the justifications that led to their development and the expected effects of each. The different elements identified were qualified as I, C, A, M, and O, and were then put together to create ICAMO configurations. As explained in the introduction, the outcomes (O) considered were the enrollment and retention of students in the TABADO program.

### 2.4 Data collection

#### 2.4.1 Multiple case study

In order to compare the initial theory with the realities in the field, we conducted a multiple case study [23]. The purpose was to collect data in different contexts to test and enrich the initial theory. A case was defined as a school benefiting from the TABADO program. The study was exhaustive as, to our knowledge, it investigated all the schools that had implemented the program in France between September 2016 and June 2019. The case study included two stages:

- an exploratory case study (three cases): its specific objective was to describe how the TABADO program was implemented in practice, to identify the main elements influencing its functioning in order to enrich the initial theory, and to build adapted investigation tools for the following case study. Here, TABADO was implemented in 2016/2017 as part of an underlying research program led by the Institut national de la santé et de la recherche médicale (Inserm) [24] in three vocational schools in the Grand Est region of France. The research coordinator was also the implementer of the program and the person in charge of the present case study (AV). The roles of researcher and on-the-ground actor were therefore combined.
- an explanatory case study (seven cases): its specific objective was to test and enrich the preliminary hypotheses from the previous stages by studying TABADO in new implementation contexts, using investigative tools designed with the findings of the exploratory case study. Here, TABADO not only included purely vocational schools, but was extended to high schools that offered vocational tracks. In total, five high schools were included in the Ile-de-France region in 2017–2018, and two vocational schools in the Nouvelle-Aquitaine region in 2016–2018. These schools were recruited in the framework of the projects led by the Ligue contre le cancer d’Ile-de-France and the Instance Régionale d’Education et de Promotion de la Santé Nouvelle-Aquitaine, respectively. Another novelty was that the program was implemented outside the original research conditions and was therefore conducted and implemented by on-the-ground actors and not by researchers. The initial protocol was thus adapted accordingly. Unlike the previous phase, the researcher (AV) was not the local implementer, but an independent observer of the program implementation.

Their respective contents are detailed in Table 1.

**Table 1:** Multiple case study: Type of investigation per case.

	Exploratory case study			Explanatory case study						
**Implementation context**	**Research**			**Real-life**						
**Type of school**	**Vocational schools**					**High schools**				
**Region of investigation**	**Grand-Est**			**Nouvelle-Aquitaine**		**Ile-de-France**				
**Case identification**	G1	G2	G3	N1	N2	I1	I2	I3	I4	I5
**Observation of environment and organization**	x	x	x	-	-	x	-	-	x	x
**Observation of information sessions**	9	8	1	-	6	5	-	-	5	5
**Observation of group sessions**	4	3	NA^a^	-	-	1	-	1	0	3
**Interviews with students:**										
** Volunteers**	10^b^	9 ^b^	NA^a^	-	-	5	3	-	-	1
** Non volunteers**	-	-	-	-	-	-	2	-	-	-
** Program drop-outs**	-	-	NA^a^			-	1	-	2	-
**Interviews with on-the-ground actors:**										
** Referent in the school**	Informal	Informal	-	-	-	1	-	1	1	-
** Tobaccologists**	Informal	Informal	NA^a^	-	-	1		-	1	
** Implementers**	NA^c^ (common to all 3 cases)			1	1	2 (common to all 5 cases)				
** Teachers**	Informal	Informal	-	-	-	-	1	3	-	-
**Inter-site seminars (feedback and validation of hypotheses)**	x	x	-	-	-	x	x	x	x	x

^a^ NA = not applicable because no student enrolled in the program

^b^ secondary use of interview transcripts from underlying research n = 16/19 [27]

^c^ NA = not applicable because only one person with the role of coordinator/researcher

In order to better understand the different facets of the program and to strengthen the validity of our results, we carried out data triangulation (interviews with the program beneficiaries and the different actors on the ground), and method triangulation (defined here as the use of multiples methods to answer a research question, i.e., observations, interviews and feedback seminars), as recommended in qualitative research [25, 26]. Triangulation aimed at identifying “regularities”, as they are referred to in realist evaluations, to develop ICAMO configurations. The resource person in charge of implementing the program in each school was designated as the "referent". The referents included professionals from various sectors: school nurse, chief education adviser, special educator, supervisor, teacher, or the boarding school prefect. The person in charge of deploying the program within the project’s supporting structure (external to the school) was referred to as the "local implementer".

*2*.*4*.*1*.*1 Observations*. Observations of the different components of the intervention were conducted (i.e., information sessions and group sessions). The researcher did not observe the individual consultations with the tobacco specialists in order to maintain confidentiality between the tobacco specialist and the students. For the explanatory case study, an observation grid was developed and included information on: the speaker (profession, gender…), the audience (number of students, classes…), the intervention and its environment (pedagogical method used, factors facilitating/hindering its progress…), student reactions and attitudes (to the different topics discussed, to the speaker/other students…), program enrollment (number, enrollment process…).

Moreover, observation of the environment and organization of the program (i.e., outside official intervention times) was conducted by in-depth immersion in the field several times a week during the intervention period [28, 29]. This allowed us to observe "behind the scenes", and thus to better understand the implementation process, the adaptations made, and the occurrence of certain events. All presumed influential elements were record in a logbook.

*2*.*4*.*1*.*2 Interviews*. The interviews were conducted face-to-face (in the schools or at the respondents’ place of work) or by telephone. They were recorded with the respondents’ consent, and were fully transcribed. The interviews from the explanatory case study were a secondary use of the interviews from the underlying research project [24, 27], with the aim of gaining insights into the beneficiaries’ program experience. For the exploratory case study, an interview guide was developed for each type of interviewee, which included information about the respondent, their view of their school, the program implementation (preparation, adaptations…), reasons for enrolling in the program/not enrolling/dropping out, reasons for quitting, their opinion on the different components of the program, suggestions for improvement, and their perception of the positive/negative effects of the program.

*2*.*4*.*1*.*3 Seminars*. Three feedback seminars were organized to bring together all the stakeholders (researchers, referents, local implementers, tobacco specialists, school nurses, principal education advisors, managers of the project’s support structures) to share experiences between schools (obstacles/levers throughout the program’s implementation), and to propose adaptations to best suit local contexts. This helped to enrich and refine the program theory hypotheses.

#### 2.4.2 Self-questionnaires

As part of the underlying TABADO research and evaluation projects, self-administered questionnaires were distributed to students who attended the information sessions and/or enrolled in the program. Questionnaires were developed by each structure in charge of the implementation of TABADO (available on request). Questions that described the students (gender, age) and their declared smoking habits (smoking status, age of first cigarette and of daily smoking, means of cigarette consumption, number of attempts to quit in lifetime, intention regarding quitting smoking) were selected for secondary use in this work.

### 2.5 Data analysis and ICAMO configurations

#### 2.5.1 Multiple case study

We conducted a directed content analysis on the data from the interviews, the logbook and the observations [30]. The main objective, to “identify the factors and mechanisms that promote/hinder the enrollment and retention of student smokers in this program,” guided the entire iterative process. The interviews from the exploratory case study were coded using the N’Vivo software and were shared between the three professionals from the underlying research project [27], according to their own field of expertise (implementation, psychology, sociology). Only the implementer’s analysis is included in this paper. The interviews from the explanatory case study were analyzed using the RQDA software. The remaining material was analyzed manually. These elements, coupled with a capitalization of the researcher’s field experience (AV), made it possible to establish hypotheses to answer the research questions. The hypotheses were then presented during the feedback seminars in order to collate them with the opinions of the different on-the-ground actors, and to confirm or invalidate them if necessary. Hypotheses were gradually established over the course of the case study (S2 Fig). At this stage, they were just part of ICAMO configurations, with some repetitive factors, or regularities, which have been highlighted.

After the last seminar, a coding table was developed using the elements identified in the initial theory and during the exploratory case study. The main themes were I, C, A, M, and O, according to the realist evaluation principles. Repetitive factors identified during the intervention and the seminars were used as subthemes of each of the main themes. The content of this table was intended to evolve as the analysis was performed. A vertical analysis was conducted first, with the aim of identifying and classifying all the items of interest case by case according to the coding table. A horizontal synthesis was then carried out, aimed at analyzing the content of each theme (I, C, A, M and O) in a transversal way across the different cases, and to establish links between the different themes. The ICAMO configurations were thus gradually constructed.

#### 2.5.2 Self-questionnaires

To describe the study population, we used aggregate data collected from the three program implementations (Grand-Est, Ile-De-France, Nouvelle-Aquitaine). Qualitative variables were expressed as percentages, quantitative variables by the mean with the standard deviation.

### 2.6 Ethical approval

TABADO was implemented in a research context in the Grand-Est region of France after obtaining the authorization of the Comité de Protection des Personnes Est III (n°ID-RCB 2016-A00317-44, dated 04/06/2016). It was implemented in a routine context in the other two regions and fell within the framework of intervention authorization for the structures concerned. In all cases, students and parents of minors were informed about, and had the possibility to object to, the collection of individual data. To enroll in the program, students and parents of minors had to sign a written consent.

The present investigation is based on secondary exploitation of the data collected in the framework of the three program implementations, completed by an *ad hoc* collection of qualitative data (interviews and observations). The data collected were anonymized and protected according to the usual procedures of the supporting structures (password, safeguards, etc.). In view of the documents at its disposal, the Research Ethics Committee of Bordeaux issued a favorable opinion for the publication of this research (Reference CE-GP-2021-25).

## 3 Results

### 3.1 Initial program theory

**Fig 1:**
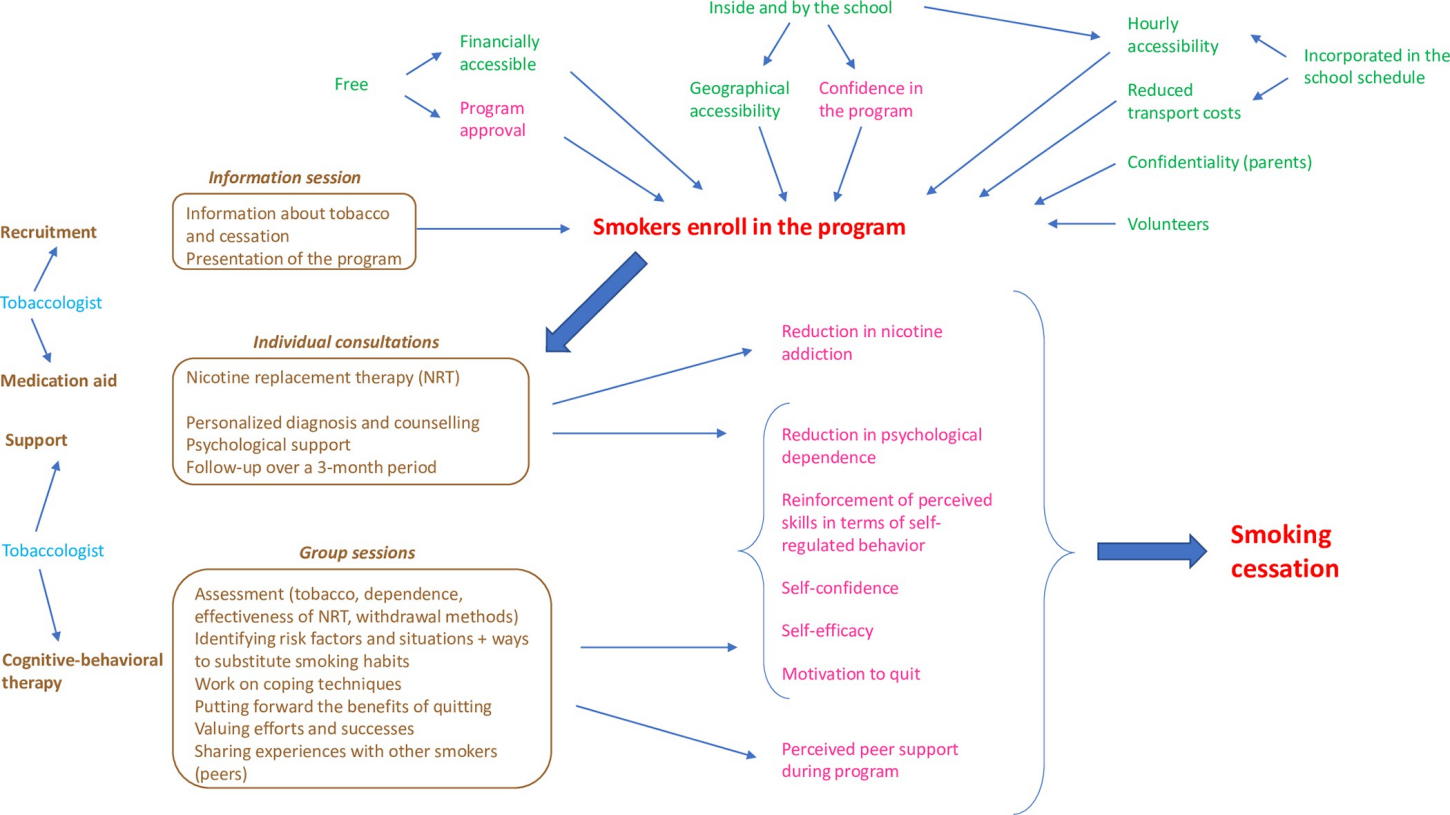
Initial TABADO theory.

The initial theory presents the different components of the intervention and the conditions that allow the activation of the main mechanisms that lead to smoking cessation. It is shown in Fig 1.

**Fig 2:**
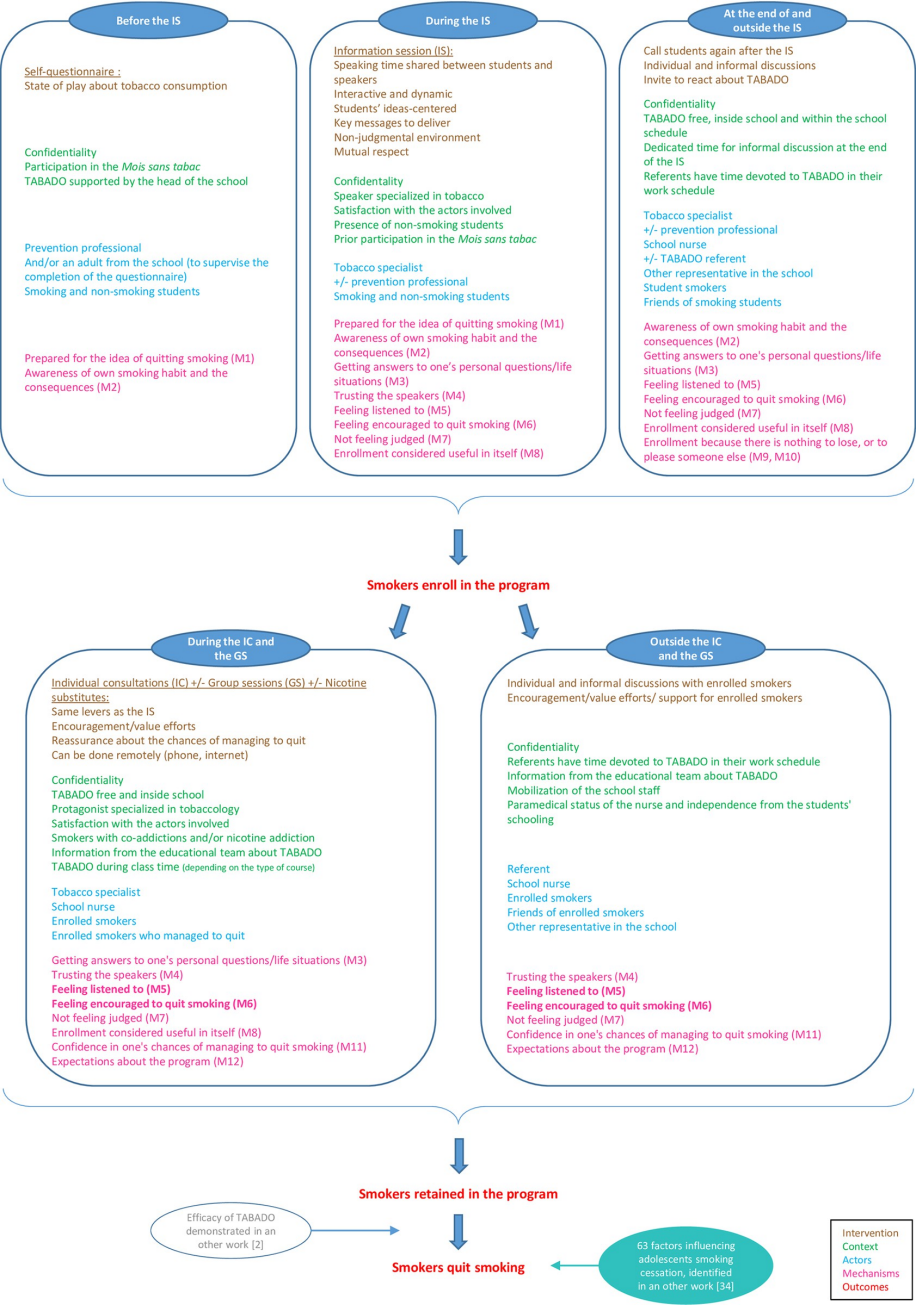
Enriched TABADO theory.

**Figs 1 and 2:** *color coding for the identification of ICAMO configurations in the program theory*

Brown: Intervention

Green: Context

Blue: Actors

Pink: Mechanisms

Red: Outcomes

The three interventional components are the information session, the individual consultations and the group sessions. The last two were conducted by the tobacco specialist. Contextual elements encouraged the smokers to enroll in the program: free access, accessibility in terms of time and place, voluntary participation and confidentiality with respect to parents. According to the designers, these interventional and contextual aspects were the most likely to trigger mechanisms that would incite enrollment in the program and, subsequently, smoking cessation.

### 3.2 Multiple case study

#### 3.2.1 Description of the cases

Ten schools were involved in the three regions, and the study included between 269 and 509 students (Table 2). The prevalence of smoking among the students included ranged from 17.1% (in high schools) to 54.0% (in vocational schools). Other school and student characteristics are shown in Table 2 (descriptive data from the N2 site in the Nouvelle-Aquitaine region were not available).

**Table 2:** Description of the cases included in the study.

	Ile-de-France	Nouvelle-Aquitaine		Grand-Est
		N1	N2	
Types of school	High schools	Vocational schools		
**Number of schools**	5	1	1	3
**Total number of questionnaires**	**509**	**134**	**135**	**300**
**Gender**			*Missing*	
*Male*	328 (64.4%)	83 (61.9%)		231 (77.0%)
*Female*	181 (35.6%)	50 (37.3%)		68 (22.7%)
*Missing*	0	1 (0.7%)		1 (0.3%)
**Age**			*Missing*	
*Mean (standard deviation)*	17.0 years (1.6)	17.0 years (1.7)		17.3 years (1.2)
*Missing*	4	4		0
**Smoker status**			*Missing*	
* Non smoker*	386 (75.8%)	61 (45.5%)		116 (38.7%)
*Ex smoker*	36 (7.1%)	11 (8.2%)		21 (7.0%)
***Smoker***	**87 (17.1%)**	**62 (46.3%)**		**162 (54.0%)**
*Missing*	0	0		1 (0.3%)
***For smokers*:**				
**Age of first cigarette**			*Missing*	
*Mean (standard deviation)*	13.1 years (2.3)	12.8 years (2.3)		13.6 years (1.9)
*Missing*	18	10		7
**Age of daily smoking**			*Missing*	
*Mean (standard deviation)*	14.8 years (1.9)	14.1 years (2.3)		14.8 years (1.5)
*Missing*	27	13		10
**Mean of cigarettes/daily consumption**			*Missing*	
*Mean (standard deviation)*	7.3 (7.1)	11.8 (7.9)		13.1 (8.9)
*Missing*	26	13		0
**Number of attempts to quit in lifetime**			*Missing*	
*Never*	24 (27.6%)	24 (40.7%)		74 (45.7%)
*At least once*	42 (48.3%)	28 (47.5%)		88 (54.3%)
*Missing*	21 (24.1%)	10		0
**Intention regarding smoking** ^**a**^			*Missing*	
*No intention to stop smoking*	31 (35.6%)	31 (50.0%)		85 (52.5%)
*Missing*	not applicable because of multiple choice question			

^*a*^ It was asked to students if they had the intention to stop smoking, and within which time period. We recoded these data to only keep the information “intention to stop smoking: yes or no”.

In total, 94 students who smoked enrolled in the TABADO program (36 in the Grand-Est, 36 in Ile-De-France, 22 in Nouvelle-Aquitaine). This number should not be compared to the number of smokers who completed the questionnaire, as some students who did not come to the information sessions and were therefore not considered as part of the target group also enrolled in the program (e.g., students from other classes in the school). The qualitative survey showed that about half of the smokers enrolled in TABADO attended the information session; the others were recruited differently (*see 3*.*2*.*2*.*1*. *Enrollment in the program*).

#### 3.2.2 ICAMO configurations

In line with our research questions, we decided to present only student-centered ICAMO configurations. However, the analysis showed that it was necessary to take into account more organizational—meso—elements related to the implementation of TABADO in the school. These are presented in other works [31, 32], and in the form of a practical guide for on-the-ground actors, available in the Santé Publique France evidence-based intervention register [3].

The configurations are presented in two main parts corresponding to our research questions: enrollment of smokers in the program, and retention of smokers in the program. Each of them have been separate in subsections which are either time periods (when some specific elements to a time period had to be highlighted) or particularly important components which were transversal to all time periods. The verbatim accounts are available in S2 Table. They have been translated from French to English.

*3*.*2*.*2*.*1 Enrollment in the program*. Table 3 presents ICAMO configurations for smokers’ enrollment in the TABADO program. The relationships between the different elements are detailed in the text.

**Table 3:** ICAMO configurations for the enrollment in the program, before, during and at the end of the information session.

	Intervention	Context	Actors	Mechanisms	Outcomes
**Before the information session**	Self-questionnaire: State of play about tobacco consumption	Confidentiality Participation in the Mois sans tabac TABADO supported by the head of the school	Prevention professional, +/- an adult from the school (to supervise the completion of the questionnaire) Smoking and non-smoking students	Prepared for the idea of quitting smoking (M1) Awareness of own smoking habit and the consequences (M2)	Smokers enroll in the program
**During the information session**	Information session (IS): Speaking time shared between students and speakers Interactive and dynamic Students’ ideas-centered Key messages to deliver Non-judgmental environment Mutual respect	Confidentiality (no longer ensured if presence of teachers at the IS) Mobilization of teachers in TABADO (reinforced if presence of teachers at the IS) Speaker specialized in tobacco Satisfaction with the actors involved Presence of non-smoking students Prior participation in the Mois sans tabac	Tobacco specialist, +/- prevention professional Smoking and non-smoking students	Prepared for the idea of quitting smoking (M1) Awareness of own smoking habit and the consequences (M2) Getting answers to one’s personal questions/life situations (M3) Trusting the speakers (M4) Feeling listened to (M5) Feeling encouraged to quit smoking (M6) Not feeling judged (M7) Enrollment considered useful in itself (M8)	
**At the end of and outside the information session**	Call students again after the IS Individual and informal discussions Invite to react about TABADO	Confidentiality TABADO free, inside school and within the school schedule Dedicated time for informal discussion at the end of the IS Referents have time devoted to TABADO in their work schedule	Tobacco specialist, +/- prevention professional School nurse, +/- TABADO referent Other representative in the school Student smokers Friends of smoking students	Awareness of own smoking habit and the consequences (M2) Getting answers to one’s personal questions/life situations (M3) Feeling listened to (M5) Feeling encouraged to quit smoking (M6) Not feeling judged (M7) Enrollment considered useful in itself (M8) Enrollment because there is nothing to lose, or to please someone else (M9, M10)	

The initial theory was that smokers would be enrolled via the information session (IS). However, the information session was only one of the entry points to the program, and enrollment was also encouraged before the meeting and outside the meeting.

Several *mechanisms (M)* came into play that led to the *enrollment of smokers in TABADO (O)*. These mechanisms acted in an interdependent and synergistic manner. To increase the chances of smokers enrolling, they had to *have been prepared for the idea of quitting smoking (M1)* prior to the presentation of TABADO. In addition, they had to *be made aware of their own smoking habit and its consequences (M2)*, *be given answers to the questions they asked themselves or to the life situations they encountered (M3)*, and they *have confidence in the speaker who presented TABADO (M4)*. They needed *to feel listened to with regard to their questions/experiences (M5)*, and *to feel encouraged in their attempt to quit smoking (M6)*. On the other hand, they needed *not to feel judged at any time (M7)*. Finally, they had to *feel that their enrollment in TABADO could be useful (M8)*; this last mechanism was derived from mechanisms M1, M2, M3 and M5, in particular. Some other mechanisms explaining enrollment were expressed more independently: *enrolling because they have nothing to lose (M9)* or *enrolling to please someone else (M10)*.

For these different mechanisms to occur, several *contextual conditions (C)* and *interventional levers (I)* had to be met.

#### Before the information session

*Awareness of their own smoking habit and its consequences (M2)* was boosted by *having students fill out a questionnaire (I)* a few weeks before the date of the IS, which allowed them to take stock of their smoking habit and gave them benchmarks (S2 Table).

In order for the students to answer honestly and really question their own consumption, *the confidentiality of the questionnaire had to be guaranteed (C)* (anonymous questionnaire, kept by the student…). This would also *prepare them for the idea of quitting (M1)*. The school’s active participation in the *Mois sans tabac (C)* prior to the start of TABADO also provided a context that reinforced these two mechanisms (*note*: The "*Moi(s) sans tabac*" campaign is an annual national event in France, which encourages the population to stop smoking for 30 days in November [33]).

#### During the information session

To encourage enrollment, students needed *to feel listened to and be able to express their opinions (M5)*, leaving the IS with *answers to questions they had about tobacco or even about co-consumption (M3)*. This gave smokers *the feeling that their enrollment in TABADO would be useful and that they would benefit personally (M8)*. Therefore, the IS should not have an academic format, *speaking time should be shared between the speaker and the students*, *and among the students themselves (I) to be participatory/interactive and dynamic (I)*. It should *be student-centered*, *focused on students’ questions*, *ideas (I)*. However, *an IS focused solely on the fun aspect (I)*, even if it appealed to the students, was not enough to get them to enroll as *it would not trigger students’ reflection*, *and thus would not raise their awareness of their own smoking habit and its consequences (M2)* (S2 Table).

Fun facilitation tools could be used, such as an interactive quiz, but each question/answer should be picked up on by the speaker for discussion and debriefing. *Key messages (I)* that were relevant to students are listed in the TABADO 2 guide, such as the composition of cigarettes, the different types of dependence, and the health effects related to physical appearance and sexuality.

In order for *a dialogue to take place between the speaker and the students (O)*, the latter *had to trust the speaker (M4)*. For this to happen, *confidentiality had to be guaranteed (C)* (*see section on Confidentiality*). Moreover, the students *needed not to feel judged (M7)*. *The speaker (A)* therefore had to be careful *not to moralize (I)* and *to ensure mutual respect among all the students (I)*.

In addition, *awareness of their own smoking habit and its consequences (M2)* was enhanced by the fact that *the IS speaker is a tobacco specialist (C)*. Indeed, even if students were aware of certain health warnings related to tobacco, they tended not to believe them (e.g., the graphic warning labels on cigarette packages). However, when *the facts were presented (I) by a speaker (A)* who is *a tobacco specialist (C)*, students *gained confidence (M4)* and thus *greater awareness of the truth behind the health warnings (O)* (S2 Table).

*Awareness (M2)* could also be promoted by *the presence of non-smoking students during an IS (C)*, who explained their experience of their classmates’ smoking (e.g., the unpleasant smells, the time spent smoking, the obsession with procuring cigarettes…) (S2 Table).

Finally, *non-smokers (A)* were not to be left out during the IS, as they could help to spread the messages and the information given, and thus *encourage their friends who smoked to enroll and not give up (M6)* (S2 Table).

In cases where *non-smokers do not feel concerned by IR (C)*, they may not listen and can be disruptive, *which hinders smokers who want to listen (O)*.

#### At the end of the information session

Students should not be expected to enroll directly after an IS, which is often dense with information, debate and discussion. Agreeing to engage in the cessation process may require additional time *to prepare for the idea of quitting (M1)*.

*Contacting the students again some time after the IS (I)* may be a good way *to trigger enrollment (O)*. When the school participated in the *Mois sans tabac (C)* before the IS, the thought of starting a cessation program may have already been initiated, and the chances of enrolling in TABADO may have increased (S2 Table).

In addition, it was essential to *keep some free time at the end of the IS (C)* to present TABADO, *then go through the rows and discuss it individually and informally with the students (I)*, especially those who said they were smokers. The idea was to *open up the discussion with an open-ended question that prompted them to react to the TABADO program; e*.*g*., *"What do you think of the program that was just presented*?*" (I)*. These individual and informal discussions both reinforced *the feeling of being personally listened to and encouraged (M5*, *M6)*, and heightened *awareness (M2)* by encouraging students to respond to the exchanges that had just taken place. In addition, such informal exchanges were more confidential than speaking in front of the whole class, and so was is *less fear of being judged (M7)*, making it easier *to address the specific and sometimes personal questions that some students had (M3)* (S2 Table).

Activating these mechanisms *reinforced the feeling for smokers that enrolling in TABADO would be useful (M8)*. Thus, when this informal time was taken, *additional students enrolled (O)*. It is even more important when *the group size is larger during the IS (C)* as the speaker cannot hear all the comments students make to each other. Without this time, students tend to leave the room as soon as the bell rings and *do not enroll (O)*. It is easier for the IS speaker to have another person to help with the informal discussions with students: e.g., *the school nurse (A)* or *the school’s referent (A)* (S2 Table).

It is also a good idea for *back-to-back IS to take place in the same room (C)* so that the speaker remains available at the end of the IS, rather than having to pack up his or her equipment and go to another room, in which case *informal exchanges cannot take place (O)*.

#### Outside the information session

Half of the enrollments came from students who did not take part in the IS, but came from *discussions initiated by the school referent and/or the nurse with students who smoked (I)*. This targeted recruitment was facilitated when *the people in the school involved in TABADO (A) knew the students well*, *and knew who was a smoker (C)* and when *they were given dedicated time for TABADO by the school head (C)* (S2 Table).

As described above, such individual and informal discussions activated many of the *mechanisms (M2*, *M3*, *M5*, *M6*, *M7*, *M8)* that *encouraged smokers to enroll in TABADO (O)*. The encouragement to join sometimes came from *friends (A)*, rather than from an adult in the school. In this sense, the *IS introduced TABADO to entire classes (I)* and activated the support that *friends (A)* could provide.

Thus, the IS was essential on several levels: to raise awareness and inform all the students about tobacco and cessation, to create information relays among peers to encourage smokers to enroll, and to directly encourage smokers to enroll.

A snowball effect was sometimes observed. When *an enrolled smoker was satisfied with his/her first TABADO consultation (C)*, (s)he would talk about it with others, *leading to new registrations (O)* (S2 Table).

#### Reasons for enrollment

The fact that the *TABADO program was completely free (C)*, *took place in the school (C)*, and was *scheduled entirely during school hours (C) facilitated enrollment and retention in the program (O)*. While some students who smoke enrolled in TABADO because they really wanted to try to quit or cut down on their smoking (the *perceived usefulness of the program (M8)* being that it would help them to achieve their goal), for others, enrollment was motivated by other reasons. For some smokers, *the reason for enrolling (M8)* was directly and solely related to missing classes. At other times, *students enrolled (O)* because *they felt they had nothing to lose (M9)*, *the program was free (C) and it was on-site at the school (C)* (S2 Table).

Sometimes they enrolled to *please a third party (M10)* (a boyfriend/girlfriend, the school nurse, a parent…). If these non-tobacco motivations did not change, students tended to *abandon the program quickly of their own accord (O)*. For others, however, a real motivation to quit smoking developed unexpectedly, and these students then *followed the program seriously (O)*. This motivation was supported by the fact that *they felt listened to (M5) and encouraged (M6) by the tobaccologist (A) and by the other smokers in the group (A)* (see 3.2.2.2 Staying in the program). It was therefore essential to *accept all enrollments in the program (I)*, regardless of the initial reason for enrolling.

#### Confidentiality

A necessary condition for the various student-related mechanisms to occur was *ensuring confidentiality of exchanges and written documents (C)*. This condition was valid as early as the IS so that *students would not be afraid to open up and to enroll in TABADO (O)*. It remained essential throughout the program *to keep smokers in the program (O)*. For example, students needed to be assured that their discussions were confidential and that their parents, teachers and employers would not be informed of their use of tobacco. When *the speaker was a health professional (C)* (e.g., school nurse, tobaccologist doctor), students were reassured by the obligation of medical confidentiality. In a confidential atmosphere, students can confide, allowing *them to obtain answers to the questions they ask themselves or to the life situations they encounter (M3)*, which then triggers *awareness of their smoking habit (M2) and a feeling of usefulness concerning TABADO (M8)*.

Since the IS were held with the whole class, teachers were sometimes invited or asked to stay during this session. Exchanges during the IS were consequently restricted by *the presence of teachers (C) as confidentiality was no longer ensured (C)*, and *students were afraid of being judged (M7)* (S2 Table).

For some students, their use of tobacco or other substances was taboo and a personal subject that they did not want to discuss in front of their teachers, or sometimes in front of any adult in the school. They were afraid to participate or ask questions, which made it *less likely that they would enroll in the program after the meeting (O)*.

This effect was observed in high schools *where smoking was often considered as taboo (C)*, but not in vocational schools where *smoking was seen as the norm (C)* and students talked about it freely, even in front of the teachers. In a climate *where smoking is considered taboo (C)*, *the various on-the-ground actors (referent*, *school nurse*, *teachers*, *etc*.*) (A)* must be particularly vigilant in their discourse so that *smokers do not feel judged (M7)*, as this would block any discussion. It should be noted that students may fear judgment from adults in the school, from other students, or from people around them outside the school (especially their families). The fear of judgment is less pronounced with people from outside the school (IS speaker, tobacco specialist). However, *the presence of teachers at the IS (C)* can have certain advantages, in particular *raising their awareness of smoking cessation and TABADO (O)*, encouraging their *acceptance of the TABADO program (O)* (the consultations took place during school hours), and making it possible for them *to act as information relays (O)*, to encourage smokers to enroll and to give them support during the cessation process.

*3*.*2*.*2*.*2 Staying in the program*. Table 4 presents the ICAMO configurations for keeping smokers in the TABADO program. The links between the different elements are detailed in the text.

**Table 4:** ICAMO configurations for keeping smokers in the TABADO program.

	Intervention	Context	Actors	Mechanisms	Outcomes
**During the individual consultations and the group sessions**	Individual consultations: Same levers as the IS Encouragement/value efforts Reassurance about the chances of managing to quit Can be done remotely (phone, internet) Group sessions: Same levers as the IS Encouragement/value efforts Reassurance about the chances of managing to quit Nicotine substitutes Individual consultations, group sessions and nicotine substitutes have to be arranged according to students’ wishes and needs	Confidentiality TABADO free and inside school Protagonist specialized in tobaccology Satisfaction with the actors involved Smokers with co-addictions and/or nicotine addiction Information from the educational team about TABADO TABADO during class time^a^	Tobacco specialist School nurse Enrolled smokers Enrolled smokers who managed to quit	Getting answers to one’s personal questions/life situations (M3) Trusting the speakers (M4) **Feeling listened to (M5)** **Feeling encouraged to quit smoking (M6)** Not feeling judged (M7) Enrollment considered useful in itself (M8) Confidence in one’s chances of managing to quit smoking (M11) Expectations about the program (M12)	Smokers retained in the program
**Outside the individual consultations and the group sessions**	Individual and informal discussions with enrolled smokers Encouragement/value efforts/ support for enrolled smokers	Confidentiality Referents have time devoted to TABADO in their work schedule Information from the educational team about TABADO Mobilization of the school staff Paramedical status of the nurse and independence from the students’ schooling	Referent School nurse Enrolled smokers Friends of enrolled smokers Other representative in the school	Trusting the speakers (M4) **Feeling listened to (M5)** **Feeling encouraged to quit smoking (M6)** Not feeling judged (M7) Confidence in one’s chances of managing to quit smoking (M11) Expectations about the program (M12)	

^a^Can become a barrier to retain smokers in the program depending on the type of course *(with an end-of-year exam/specific to technical learning/particularly enjoyed) as students get worry about repeatedly missing these classes (M13) and eventually drop out of TABADO (O)*. Therefore, *the referent (A) must anticipate the scheduling of TABADO in relation to the end-of-year exam schedule (I)*, *and be careful to not always impact the same course/teacher (I)*.

The M3 to M8 mechanisms presented for program enrollment apply equally to individual consultations (IC) and group sessions (GS) *to keep smokers in the program (O)*. That is, throughout the program, young people needed *to feel that they could trust the tobaccologist (M4)*, that (s)he would give them *answers/advice tailored to their own questions and life situations (M3)*, *listen to their experiences (M5)*, *and encourage them to quit (M6) without them feeling judged (M7)*. Finally, when they felt that their *participation in TABADO was personally useful (M8)*, they tended to *continue the program until the end (O)*. However, two mechanisms were much stronger than the others in this phase of the program: *the feeling of being listened to (M5)* and *the feeling of being encouraged (or more precisely "valued" at this stage) (M6)*. They were also linked to a new key mechanism during this phase: *confidence in the chances of managing to quit smoking (M11)*. Two other mechanisms were important in keeping students in the program: *expectations about the program (M12) and worry about regularly missing classes (M13)*.

The contextual conditions related to all of the already known mechanisms remained the same for keeping smokers in the program (confidentiality, free access, tobacco specialist, etc.), with the exception of the *scheduling of intervention times during class time (C)*, which presented an additional specificity that will be detailed below. The intervention levers that applied to the IS applied equally to IC and GS (e.g., no moralizing, enough speaking time for students…).

#### The need for personalized and continual follow-up

The two main follow-up components (IC and GS) did not have quite the same function. The IC had a more intimate function; students could confide in the tobaccologist about more intimate issues related to their tobacco use or other products, or their life context, and *get personalized answers (M3)*. The GS allowed students *to share their experiences (M5) and support each other (M6)*. These two components are complementary, and it is best for students to participate in both (S2 Table).

But for some students, talking in a group or being alone in a consultation with an unknow adult was not possible. *They should not be forced to participate in either (I) since they could abandon the program (O)*.

In addition, *the duration of the IC (I)* needed to be adapted to each student: some young people may *have co-addictions (C) and/or a physical dependence on nicotine (C)*, requiring the introduction of a substitution treatment. *The tobaccologist (A)* who dealt with the students needed to determine the duration of the consultations necessary for each student, so that each one benefited from a treatment corresponding to his or her needs. This helped students to increase *their confidence in their chances of successfully quitting smoking (M11)*, thus *avoiding program drop-outs and/or cessation failure (O)*.

Regarding the time between each IC or GS, a rapid follow-up was recommended at the beginning, which could be spaced out later. The students’ schedules, *alternating between attendance at school or at their employer’s (C)*, did not always allow for the desired scheduling of intervention times. However, the most important thing was to ensure a follow-up. If it could not be done physically at the school, *a remote consultation was proposed (telephone*, *web conference) (I)* (S2 Table).

*The school nurse (A)* played a pivotal role in TABADO. For the tobaccologist, (s)he represented a colleague trained in health, who knew the students’ medical records and who therefore provided precious assistance in *adapting the care of enrolled smokers to each personal situation (O)* during follow-up. Moreover, (s)he was a *close contact for the smokers between two appointments with the tobaccologist (O)* or even *after the TABADO program ended (O)* (advice and/or delivery of nicotine substitutes), giving the students the possibility *to talk about their difficulties (M5)* and *encouraging them to continue their cessation process during difficult moments (M6)*, even in the absence of the tobaccologist (S2 Table).

For the students, *the nurse’s paramedical status (C)* and *independence from their schooling (C)* was a factor *that fostered trust (M4)* and therefore *promoted discussion on topics related to the use of products such as tobacco (O)*. Through their various roles, nurses promoted *enrollment in the program (O) and the upkeep of cessation efforts (O)*.

#### Confidence in the chances of success of quitting smoking

*Confidence in their chances of quitting (M11)* encouraged students *to stay in the program and to continue trying to quit (O)*. This mechanism was greatly *enhanced when a student in the follow-up group (A) successfully quit smoking (C)*, proving to his or her peers that it was possible to stop (S2 Table).

Conversely, if a student *thought (s)he had no chance of successfully quitting smoking (M11)*, this *blocked any attempt to participate in the program (O)*. For these students in particular, it was important *to help them to gain confidence in their chances of quitting successfully (I)*.

*Confidence in the chances of quitting (M11)* also strongly interacted with *feeling listened to about smoking (M5) and feeling encouraged and supported by the tobacco specialist (M6)*. While any support can help motivate students, this was particularly noticeable *when the support came from the tobacco specialist (C)*. It was thus extremely important for *the tobaccologist (A) to encourage the students (I)*, *and to value every effort and minor success (I)* (S2 Table).

It is also important to note that when *students’ expectations about the program were too high (M12)* (e.g., thinking that in one consultation they would manage to quit smoking), they *quickly dropped out (O)* as they were disappointed that the program did not meet their expectations.

#### Life priorities

The mechanisms and conditions that promoted enrollment and staying in the program may have been counterbalanced by other mechanisms related to life priorities. Indeed, some students reported being *worried about repeatedly missing certain classes (M13)*. These were *courses for which there was an end-of-year exam (C)*, *courses specific to technical learning for their future career (C)*, *or courses that they particularly enjoyed (C)*. These students sometimes preferred to miss their TABADO appointment rather than the course in question, and therefore *did not attend the full program (O)* (S2 Table).

In this context, *the scheduling of intervention time during class time (C)* was sometimes a barrier to keeping students in the program. This was reinforced by *teachers (A)* who, especially if *they were not informed or were ill-informed about the TABADO program (C)*, refused to let students out of class, their goal being their students’ academic success.

This was especially noticeable at the end of the school year due to *the end-of-year exams (C)*. *The referent in the school (A)* therefore had to take care *to plan the different intervention times to fit in with the courses (without end-of-year exams*, *not during practice times) (I)*, *varying them so as to not always impact the same teacher or the same course (I)*. In addition, the referent had to be careful *to start TABADO early enough in the school year (I)* so that the program did not overlap with end-of-year exam periods.

### 3.3 Refined program theory

The revised TABADO theory includes all the results described above. A synthesis of the theory is described in Fig 2. Compared to the initial theory, interventional levers have been added for each component of TABADO. All these levers are detailed in the TABADO 2 implementation guide [3]. The required contextual conditions foreseen in the initial theory were confirmed (notably the need for confidentiality), and new ones emerged. However, one of the initial conditions was shown to be a barrier to keeping students in the program at times (scheduling TABADO during school hours). Concerning the on-the-ground actors involved in TABADO, they are far more numerous and varied than in the initial theory. Indeed, the tobacco specialist was the only one who appeared originally. However, our analysis shows that it is essential to open up the program and involve a multitude of actors to encourage the enrollment and retention of adolescent smokers in the program. Finally, strong new mechanisms emerged from our analyses, such as awareness of one’s own smoking and its consequences, the need to feel listened to, and the fear of judgment. Other mechanisms had already been identified but have been clarified, such as the perception of support that is not specific to peers but can also be that of the tobaccologist or another adult in the school, or confidence in one’s chances of success at quitting that is reinforced if another student in the group has managed to stop. In contrast, two mechanisms that were initially predicted did not emerge in our analysis: a reduction in the psychological dependence on cigarettes, and the reinforcement of perceived skills in terms of self-regulated behavior.

## 4. Discussion

### 4.1 Summary of findings

This work helps to explain why adolescent smokers enroll and stay in a school-based smoking cessation program such as TABADO. In addition, we showed that we need to consider TABADO in a more comprehensive way than was done with the original TABADO research protocol:

- through the diversity of the on-the-ground actors involved (the tobaccologist, of course, but also the school nurse, the teachers, the smokers’ friends…),
- by the program’s continuity, even outside official intervention times (i.e., outside of the information session, individual consultations and group sessions)
- and by collaborating with existing national strategies (e.g., the *Mois sans tabac*).

In this respect, TABADO should be considered less as an isolated intervention, and more as a global strategy for the school. A practical guide has been produced for those working in the field to implement this new strategy, optimized and adapted to real-life conditions: the TABADO 2 guide [3, 32].

In addition to factors influencing enrollment and staying in the program, 63 factors directly influencing adolescent smoking cessation were identified in a systematic literature review and are presented elsewhere [34]. Half were psychosocial factors (e.g., self-efficacy in quitting smoking, negative beliefs about smoking) or social influences factors (e.g., friends smoke, parents smoke). They can be added to the TABADO program theory (Fig 2).

### 4.2 Strengths, limitations and future directions

The realist evaluation methodology allowed us to accompany the TABADO scale-up process in real time. Indeed, the hypotheses from the program theory were continuously readjusted and enriched thanks to new data from the field. At each point in our research, we were able to provide decision-makers with evidence to inform their choices regarding the national scaling-up of TABADO. The research method allowed us to respond to needs in the field without waiting for the study to be completed and/or this article to be published.

Another strength of our work lies in the different levels of perspective on the implementation of the TABADO program. Indeed, the researcher’s position varied according to the case study, ranging from research coordinator to outside observer. We also used data triangulation and methods triangulation, allowing us to develop a reflexive stance, and to get as close as possible to the complexity of reality [35].

The ten cases in our study provided a variety of contexts, with different populations and different school situations. However, we do not claim to be exhaustive in this respect. For example, we were only able to interview a few students who smoked and did not want to enroll in TABADO (n = 2) or who had dropped out of the program prematurely (n = 3), as these profiles were less willing to take part in a research interview. Thus, the mechanisms related to not enrolling or dropping out of the program were probably less well explored than those related to enrollment and staying in the program. Similarly, teachers who were not supportive of TABADO did not respond to our invitation to be interviewed, so we were unable to explore some of the barriers. In addition, the investigations were uneven among the ten cases, due to logistical constraints and the availability of the various stakeholders. Some cases were therefore studied in greater depth, and their results may be more extensively represented in the program theory than others. However, the evaluation of the national scaling-up will provide the study with new large-scale contexts [4]. As with the realist evaluation cycle, our refined theory may be enriched by these new experiences.

### 4.3 Comparison with the existing literature

#### 4.3.1 Methodological consideration: A new understanding of the key functions of an intervention

The ICAMO configurations resulting from this work illustrate the strong link between the components of an intervention, the implementation methods and the environment in which the intervention is implemented. This environment is governed by a multitude of factors: numerous stakeholders with equally varied attitudes and reasoning, organizational and financial constraints, a well-defined legislative framework [36]. The intervention can thus be considered as an event in the history of this complex system [37]. It modifies the system with the implementation of new activities, the creation of partnerships, the evolution of individual representations, and changes in behavior. Some authors argue that the intervention is itself an interventional system [38]. By interweaving these different concepts, we arrive at a conceptualization of interventions as systems within systems, with permeable limits between them, making it possible to take in the full complexity of reality. The interest of the ICAMO configurations is that they take this complexity into account by seeking to identify the different elements in these systems (Intervention Context Actors Mechanisms Outcomes) and to connect them with each other. Thus, in our opinion, they could be a useful tool to unpack the "black box" of complex intervention [39] and to operationalize the concept of "key (or core) function" [40, 41] for which the application remains unclear or restricted to Mechanisms alone. Thus, a key function may not be simply the mechanism, but the entire ICAMO configuration. This idea of defining the key function as an ICAMO configuration is, to our knowledge, new, and deserves to be further explored through conceptual and methodological research.

However, an intervention should not be considered as a fixed system to be reproduced identically in all intervention contexts. Indeed, adaptation may be necessary for various reasons: to culturally appropriate a program for a new target population, enhance acceptability and local commitment, increase satisfaction with the intervention, or improve the ease and feasibility of implementation with the local context [42, 43]. In most articles, adaptations are defined as planned processes to modify an intervention in order to fit it into a new context [43]. The challenge is thus to be able to make these adjustments without altering the key functions of the intervention. Certain tools have been developed to guide the on-the-ground actors. For example, The Framework for Reporting Adaptations and Modifications-Enhanced (FRAME) allows us to describe and characterize the adaptations made [44]. The Model for Adaptation Design and Impact (MADI) is more explanatory and is designed to understand the impact of the adaptations on the results, and to guide the decision-making process of these adaptations [45]. Further conceptual research by the Medical Research Council is in progress to better define the concept of adaptation and its issues, and to develop methods to operationalize the fidelity of the intervention functions [46]. The leads given by the authors at this stage point mainly towards realist evaluation, which supports our position on the use of ICAMO configurations to determine the key functions of a complex intervention.

Finally, this study shows that we can encourage smoking cessation among adolescents in a school environment if the contextual elements are taken into account to tailor the intervention to the context in which it is implemented.

#### 4.3.2 Comparison with others school-based smoking cessation programs

To our knowledge, there is no other published work on school-based smoking cessation programs using realist evaluation. However, some realist evaluation protocols in school settings aiming at reducing smoking among adolescents have been recently published [47, 48]. Their results will be particularly worthy of comparison with the present research. Most notably, if some components among the mechanisms or implementation facilitators in their program theory are the same as ours, they could be significant elements to have in all school-based smoking cessation or prevention programs, to ensure their effectiveness in real-world settings.

In other school-based smoking cessation programs that are not using realist evaluation, some results are in step with ours. A phenomenological study in Korea suggested that getting students to self-reflect (corresponding to our mechanisms M1, M2, M3) can change their smoking habits. Plus, as well as increasing self-efficacy to quit smoking (our mechanism M11), increasing self-efficacy and self-esteem more generally in life may strengthen the motivation for adolescent smokers to quit smoking [49]. On ensuring a more effective implementation, another qualitative study in Korea highlighted that the program must be supported by the head of the school and be institutionalized in the school, and must involve the school staff and non-smoking students in the program [50]. These elements are also part of our ICAMO configurations, as actors and contextual components.

## Conclusions

TABADO is now theory-based and its main key functions have been identified. Using this theory, an optimized intervention has been created, TABADO 2, which can now be transferred on a large scale and in real-life settings. This research led to the development of a guide for policymakers and practitioners on how to scale up the intervention.

## Supporting information

S1 Table List of items to be included when reporting realist evaluations. Completed RAMESES II checklist for this article.
(DOCX)

S2 Table Verbatim accounts from the interviews and the observations. (DOCX)

S1 Fig Diagram of the process for developing the TABADO program theory. (PDF)

S2 Fig Analysis cycle for the development of the TABADO program theory. (PDF)
