# Supplementary material for: Ensuring that a school-based smoking cessation program for adolescents is successful: A realist evaluation of the TABADO program and the program theory
Source: PLoS One. 2023 Apr 6;18(4):e0283937. doi: 10.1371/journal.pone.0283937 (PMC10079096; doi:10.1371/journal.pone.0283937)
Supplement: S2 Table — (DOCX) [file pone.0283937.s002.docx]

**S2 Table. Verbatim accounts from the interviews and the observations.**

All verbatims have been translated from French to English by the authors.

|  | **ENROLLMENT IN THE PROGRAM** |
| --- | --- |
| **Before the information session** | Enrolled smoker student interview: *"[The questionnaire] really makes you realize everything you do in detail, because in general when you smoke it's usual, so even if you think about quitting or things like that, well you don't think about all the details, etc. But when you do the questionnaire you realize everything, and you're in a more intense state. When you do the questionnaire, you realize everything, and you're like… It's powerful. Well, for me personally, that's what it was."* |
| **During the information session** | Nurse Interview: *"The students took it as a game. But... not as an approach to get into a project, they were too focused on the game. [...] Ah well, they found it really fun. "Yeah, we played well"! That's it, "we played well".*"  Fieldnote: "*[an electronic quiz system is used, students answer individually or in pairs and the results are displayed for all]. The speaker gives the instruction that the faster you answer the quiz, the more points you get. The students were happy to play. It goes very fast, the questions follow one another, some students don't have time to answer the questions. The students' main concern seems to be winning the game.*"  Drop-out smoker student interview: *"Honestly, the pictures on the packages are clearly useless. [...] Well, it's a photo, but anyone can put a photo of someone with a hole in the throat ... There are lots of people who don't believe the pictures. But then I know that there are some for sure [...] it happens to us, but there are others I find hard to believe. [...] There, I believe it because it's your job, you've done your research, you know what tobacco is*.”  *Enrolled smoker student interview: "We could all take part in the discussion, even those who don't smoke could ask questions, and they can help us answer because they see it from the outside. When we talked about the smell, things like that, we don't notice it because we're in it every day, they notice the smell of the cold tobacco we have on us. They know if it’s more or less pleasant [...]. And so it's a rather interesting point of view that we don't necessarily appreciate otherwise."*  Fieldnote: "*After showing the video, a non-smoker called out to a classmate, "B, aren't you disgusted with cigarettes with all this?!" At the end, when the speaker introduced TABADO, non-smokers challenged a classmate "So, you're not going to enroll?*" |
| **At the end of the information session** | Enrolled smoker student interview: *"But, uh, when we did the Mois sans tabac, we’d already been told about TABADO, but at that time we weren't really interested because we weren't really motivated to quit completely. And then we had the information session, and I know that personally it motivated me a lot, and so I encouraged her (her friend) and we signed up."*  Fieldnote: "*[Group of 27 students] The girls next to me seem to be smokers, they’re talking to each other about what the speaker is presenting and have a question that hasn't been addressed. I reassure them that it's a good question and encourage them to ask it. They don't. [...] Before the bell, some free time is dedicated to talking about the program. The girls who did not dare speak up in the group take advantage of this time to put their questions to the speaker individually."*  Nurse interview: "*We did like that in front of the students, telling them, well, those who’re interested can come. And in fact, there was little enthusiasm. So we started again, we split into two (the speaker and the school nurse), and one of us waited at the exit and the other went along the row and we said, well, what did you think of it, etc.? And in fact, this allowed us to break the ice, to approach the situation individually. We went from a class group to individuals. And we managed to get two or three students interested like that.*" |
| **Outside the information session** | Enrolled smoker student interview: "*We (the student and her friend) were actually picked up by the supervisor (the referent) because she took part in the Mois sans tabac, so she knew that our goal was to quit smoking. So she immediately told us, well sign up.*"  Fieldnote: *"[Student 2] asked to enroll in the program on the advice of [Student 1] who had enrolled the day before. Then, after the first consultation, [student 2] asked if [student 3] could enroll as well (student 3 was seen in an information session in a class with 0 enrollees, even though they appeared particularly involved and even stayed around us at the end to talk some more). Finally [student 4] wanted to enroll later (at follow-up 1) on the advice of [student 2]."* |
| **Reasons for enrollment** | Enrolled smoker student interview: *"I signed up because I thought let's try it, maybe it will help me, and they told me that maybe we could [...] try substitutes for free and uh, and I thought that's interesting because substitutes are things that I wouldn't try if I had to pay for them."*  Enrolled smoker student interview: “***Interviewer:*** *So why did you join the program?* ***Interviewee:*** *Well, my classmates encouraged me to do it because they told me that I wasn't losing anything, so, uh, so I signed up and it paid off.*" |
| **Confidentiality** | Non-smoker student interview: *"I mean, there are lots of students who want to talk, but they don't dare to talk [...], ask a lot of questions about smoking, drugs, all that stuff. [...] Because often, young people don't want to talk about it in front of the teachers because they're afraid that the teachers will talk to their parents or stuff like that."* |
|  | **STAYING IN THE PROGRAM** |
| **The need for personalized and continual follow-up** | Enrolled smoker student interview: *"I actually like both, group and personal, because I think that, personal consultations focus on us, on our different personalities, our different situations and everything. And so we get more advice that can help us. But in a group, it's great too. I really like it because we can exchange ideas."*  Tobaccologist interview: *"This is what the Haute Autorité de Santé recommends, everything should go fairly quickly, that is, we should be able to see the students at one-week intervals. [...] With school vacations, strikes, exams... it's not going well. I haven't seen [student's name] for three weeks now, so it's not going well. So I gave him a Skype session, which was very useful and worked well."*  Fieldnote: "*The school nurse is present at the group session (led by the tobaccologist). He is not in a position of official intervener, but intervenes anyway in addition to the tobaccologist. At the end of the session (it is the last one), the nurse lets the students know that he is available to continue helping them in their cessation efforts."* |
| **Confidence in the chances of success of quitting smoking** | Enrolled smoker student interview: *"Well yeah, like [name of another enrolled student], she quit smoking. That surprises me and frankly, if she can do it, I can do it too."*  Enrolled smoker student interview*: "****Interviewer:*** *So what did you think of the individual consultations?* ***Interviewee:*** *Well it was good because she listened to us, and every time I reduced my cigarette consumption, she encouraged me, so that made me want to go on."* |
| **Life priorities** | Enrolled smoker student interview: *"****Interviewer:*** *You didn't take part in the group sessions?* ***Interviewee:*** *No, it was because it was during my sports practice, and for what I’m doing next year, well I needed it actually."* |
