# Supplementary figures and images for: Ensuring that a school-based smoking cessation program for adolescents is successful: A realist evaluation of the TABADO program and the program theory
Source: PLoS One. 2023 Apr 6;18(4):e0283937. doi: 10.1371/journal.pone.0283937 (PMC10079096; doi:10.1371/journal.pone.0283937)

S1 Fig. Diagram of the process for developing the TABADO program theory.

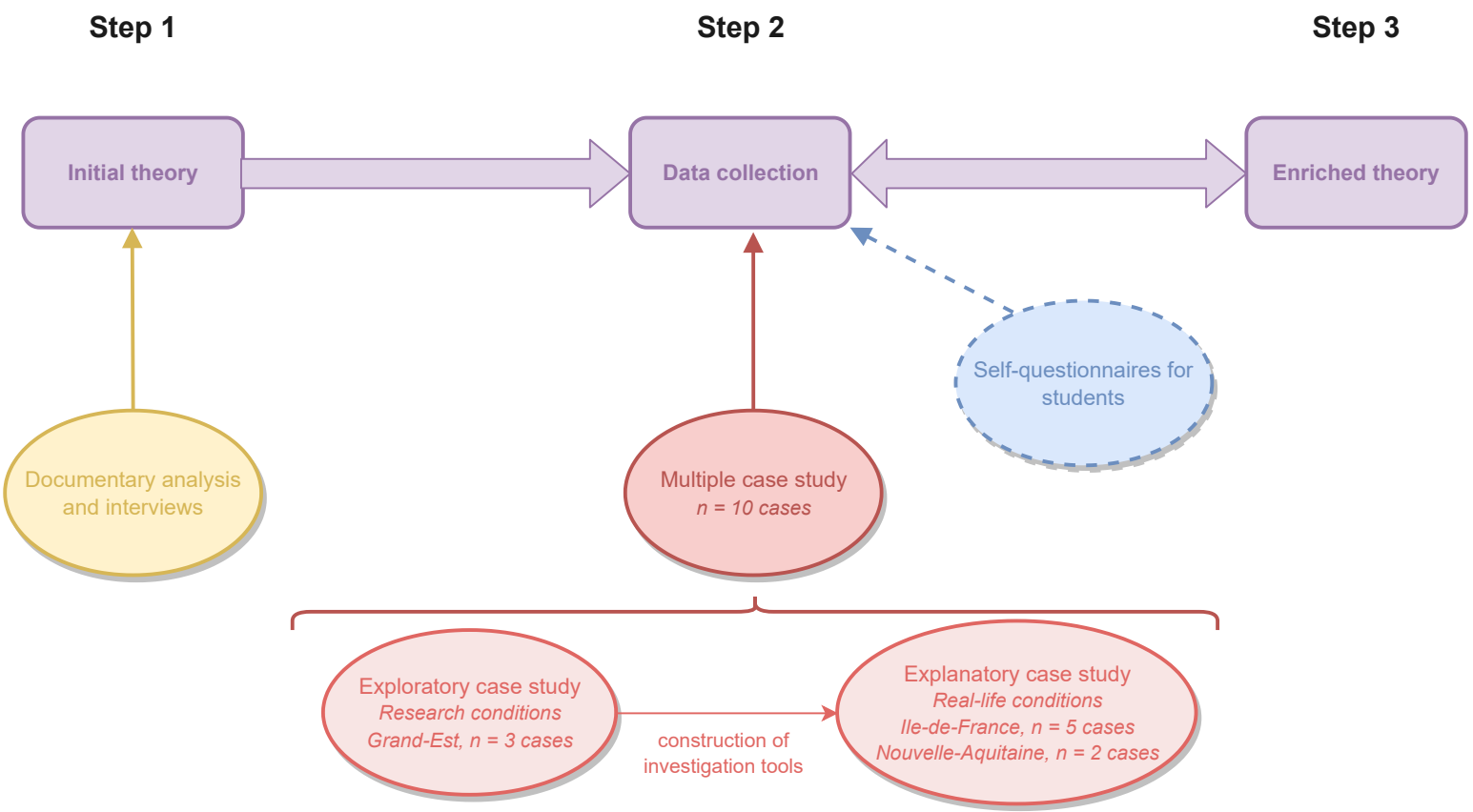

Supplement: S1 Fig — (PDF) [file pone.0283937.s003.pdf]

S2 Fig. Analysis cycle for the development of the TABADO program theory.

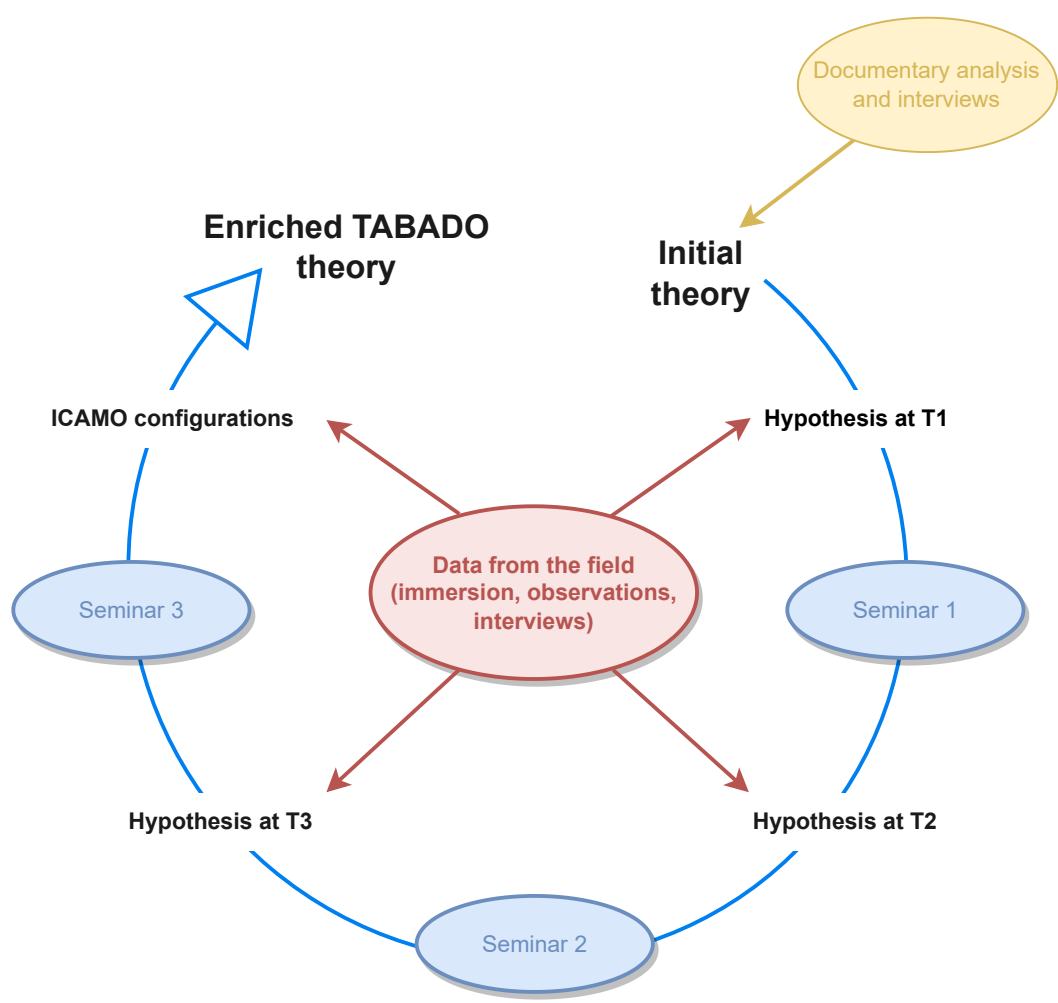

Supplement: S2 Fig — (PDF) [file pone.0283937.s004.pdf]
